# Cost-effectiveness of an integrated 'fast track' rehabilitation service for multi-trauma patients involving dedicated early rehabilitation intervention programs: design of a prospective, multi-centre, non-randomised clinical trial

**DOI:** 10.1186/1752-2897-3-1

**Published:** 2009-01-30

**Authors:** Sevginur Kosar, Henk AM Seelen, Bena Hemmen, Silvia MAA Evers, Peter RG Brink

**Affiliations:** 1 Rehabilitation Foundation Limburg, Hoensbroek, The Netherlands; 2 Department of Rehabilitation Medicine, Faculty of Health, Medicine and Life Sciences, Research School Caphri, University of Maastricht, Maastricht, The Netherlands; 3 Department of Health Organization, Policy and Economics, Faculty of Health, Medicine and Life Sciences, Research School Caphri, University of Maastricht, Maastricht, The Netherlands; 4 Trauma Centre Limburg, Academic Hospital Maastricht, Maastricht, The Netherlands

## Abstract

**Background:**

In conventional multi-trauma care service (CTCS), patients are admitted to hospital via the accident & emergency room. After surgery they are transferred to the IC-unit followed by the general surgery ward. Ensuing treatment takes place in a hospital's outpatient clinic, a rehabilitation centre, a nursing home or the community. Typically, each of the CTCS partners may have its own more or less autonomous treatment perspective. Clinical evidence, however, suggests that an integrated multi-trauma rehabilitation approach ('Supported Fast-track multi-Trauma Rehabilitation Service': SFTRS), featuring: 1) earlier transfer to a specialised trauma rehabilitation unit; 2) earlier start of 'non-weight-bearing' training and multidisciplinary treatment; 3) well-documented treatment protocols; 4) early individual goal-setting; 5) co-ordination of treatment between trauma surgeon and physiatrist, and 6) shorter lengths-of-stay, may be more (cost-)effective.

This paper describes the design of a prospective cohort study evaluating the (cost-) effectiveness of SFTRS relative to CTCS.

**Methods/design:**

The study population includes multi-trauma patients, admitted to one of the participating hospitals, with an Injury Severity Scale score > = 16, complex multiple injuries in several extremities or complex pelvic and/or acetabulum fractures. In a prospective cohort study CTCS and SFTRS will be contrasted. The inclusion period is 19 months. The duration of follow-up is 12 months, with measurements taken at baseline, and at 3,6,9 and 12 months post-injury.

Primary outcome measures are 'quality of life' (SF-36) and 'functional health status' (Functional Independence Measure). Secondary outcome measures are the Hospital Anxiety & Depression Scale, the Mini-Mental State Examination as an indicator of cognitive functioning, and the Canadian Occupational Performance Measure measuring the extent to which individual ADL treatment goals are met. Costs will be assessed using the PROductivity and DISease Questionnaire and a cost questionnaire.

**Discussion:**

The study will yield results on the efficiency of an adapted care service for multi-trauma patients (SFTRS) featuring earlier (and condensed) involvement of specialised rehabilitation treatment. Results will show whether improved SFTRS logistics, combined with shorter stays in hospital and rehabilitation clinic and specialised early rehabilitation training modules are more (cost-) effective, relative to CTCS.

**Trial registration:**

Current Controlled Trials register (ISRCTN68246661) and Netherlands Trial Register (NTR139).

## Introduction

In the Netherlands, with a population of approximately 16 million people, every year about 99.000 persons are admitted to hospital after an accident, whereas 880.000 people visit the Accident & Emergency department (A&E) after an accident [1]. These accidents lead to considerable societal costs. Direct medical costs are estimated at 1 billion Euro/year, i.e. 3–4% of the total Dutch health care budget. Production losses due to acute trauma are estimated at 4 billion Euro, thus widely surpassing costs of chronic illness like cardiovascular diseases and cancer [2-4]. Many of the patients have multiple fractures. Major causes of multi-trauma are traffic accidents, accidents at work, (extreme) sports, falls, blasts, etc. [5]. The legs (incl. pelvis) are most frequently injured in multi-trauma [6-9]. Multi-trauma occurs more often in males and in younger adults [5]; [10]. Many patients are at an age where they have a paid job. Furthermore, the rehabilitation of the multi-trauma patients may take a long time. The societal impact of multi-trauma is therefore large.

Medical care for trauma patients is a combined responsibility of hospitals, ambulance services, trauma centres, rehabilitation clinics and GHOR (medical assistance after accidents and disasters). This co-operation between teams of specialists is called 'trauma care chain' (TCC). In the present study multi-trauma is defined as having 2 or more injuries of which at least 1 is life threatening, including trauma with an Injury Severity Scale (ISS) score > = 16; complex multiple injuries on both lower extremities; a combination of 1 upper and 1 lower extremity injury, the latter of which can not be used in load-bearing; or complex pelvis/acetabulum fractures. Several tools for rating trauma severity have been designed [11]. The ISS [12,13] is used most often.

In conventional multi-trauma care service (CTCS) each of the partners has its own more or less autonomous treatment perspective, depending on the professional's individual treatment views and experience. Clinical evidence, however, suggests that an integrated multi-trauma rehabilitation service approach or 'Supported Fast track multi-Trauma Rehabilitation Service' (SFTRS), featuring:

1) Earlier involvement of the rehabilitation physician in the hospital;

2) shorter stay in hospital and earlier transfer of multi-trauma patients to a specialised trauma rehabilitation unit;

3) an earlier start of both specific physical and psychosocial treatment provided by a multidisciplinary team;

4) early individual goal setting;

5) early start of psychological and social counselling;

6) an integrated co-ordination of treatment between the trauma surgeon and the rehabilitation physician, and

7) a shorter stay in a trauma rehabilitation unit

may be more (cost-) effective. Such SFTRS approach may lead to less secondary complications associated with immobilisation, which would negatively influence recovery and quality of life. Early personalised goal setting and early treatment of depression are known to positively affect outcome. SFTRS may lead to faster reintegration into society. Early return to work and active support from the multidisciplinary rehabilitation team will lead to a more stable social network, and the patient becoming less reliant on professional care in the long term. Also, the SFTRS may reduce the length of stay of multi-trauma patients in a hospital. Furthermore, earlier rehabilitation treatment in a specialised rehabilitation unit may also reduce the length of stay in the rehabilitation clinic, thus reducing costs of hospital/clinic consumption. At this moment it is not possible to make a precise calculation of these savings. Since earlier discharge also means that patients take part in society and work earlier, costs related to production losses and patient & family costs are expected to be lower.

The main objective of this study is to examine the effectiveness, costs and cost-effectiveness of an integrated care service for multi-trauma patients, called 'Supported Fast track multi-Trauma Rehabilitation Service' or SFTRS.

The general research question is:

Which of 2 rehabilitation services, i.e. 'Conventional multi-Trauma Care Service' (CTCS) or 'Supported Fast track multi-Trauma Rehabilitation Service' (SFTRS), is most (cost-) effective from a societal point of view?

Sub-questions are:

- What are the effects of the SFTRS on generic quality of life in multi-trauma patients as compared to the CTCS?

- What are the effects of the SFTRS on functional health status as compared to the CTCS?

- What are the costs to health care and to society of the SFTRS as compared to the CTCS?

- What is the cost-effectiveness of the SFTRS as compared to the CTCS?

The general hypothesis of this study is that SFTRS is more (cost-) effective than CTCS.

## Methods

### Design

**Figure 1:**
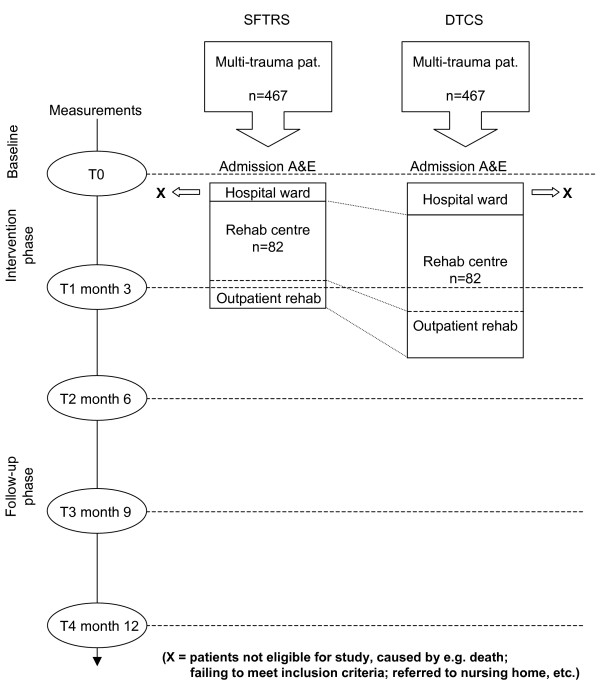
**Flow chart of the study**. SFTRS: Supported Fast track multi-Trauma Rehabilitation Service; CTCS: Conventional multi-Trauma Care Service; A&E: Accident and Emergency department.

This study is a prospective, multi-centre, non-randomised clinical trial in which two multi-trauma rehabilitation services will be contrasted, i.e. 'Conventional Trauma Care Service' (CTCS) and 'Supported Fast track multi-Trauma Rehabilitation Service' (SFTRS). The patients will be followed for 12 months (see flow chart, Figure 1). The inclusion time is 19 months.

Prior to participation, informed consent will be obtained from all participants.

All protocols used in this study have been approved by the Medical Ethics Committee of the Rehabilitation Foundation Limburg in Hoensbroek, the Netherlands.

This study is registered at the Current Controlled Trials register (ISRCTN68246661) as well as at the Netherlands Trial Register (NTR1391).

### Population

Multi-trauma patients admitted to one of the accident & emergency departments (A&E) of the participating hospitals are included. Multi-trauma is defined as having at least 2 or more injuries of which at least 1 is life threatening, including a) trauma with an Injury Severity Scale score ISS > = 16, b) complex multiple injuries on both lower extremities, c) a combination of one upper and one lower extremity injury, the latter of which can not be used in load-bearing, or d) complex pelvis/acetabulum fractures (for in- and exclusion criteria see Table 1)

**Table 1:** Inclusion and exclusion criteria

Inclusion criteria	Age > = 18 years
	Multi-trauma (as defined above)
	Hospitalisation after A&E admission
	Rehabilitation indication, i.e. lasting impairments or handicaps are expected
	Adequate Dutch language skills
Exclusion criteria	Severe alcohol and/or drug abuse
	Severe psychiatric problems

A&E: Accident and Emergency room

Participants for the SFTRS will be recruited in the adherence of the trauma centre in the south of the Netherlands. The reference group will be recruited in the adherence of one of the other trauma centres in the Netherlands. The inclusion of the participants will start in October 2008 and will continue until May 2010. Both medically and ethically it is not feasible to randomise the acute multi-trauma patients across the two trauma centres mainly due to geographical distances. Therefore, a non-randomised controlled clinical trial is used.

Based on the 2005–2007 databases from the participating centres, the influx numbers for the SFTRS group and the CTCS group are expected to amount to about 364/year and 370/year respectively. Prospective data from a pilot study in 2008 showed that, within 3.5 months, in each of the two centres 86 multi-trauma patients were admitted to the A&E. Given an inclusion period of 19 months, approximately 467 patients in each of the respective regions are expected, totalling 934 for this study (see flow chart, Figure 1).

To date, no exact data on differences in quality of life outcome between different treatment services for multi-trauma patients are available. Czyrny and co-workers [14] report an improvement in motor FIM score of 30.2 in a small group of bilateral lower limb multi trauma patients having received both hospital and subsequent rehabilitation treatment, at a mean length of stay of 62.8 (+/-6.0) days. In order to detect a difference of 15% of such improvement in FIM score between SFTRS and CTCS at T1 (assuming a two-sided significance level of 0.05, a power of 80%, and a common standard deviation (sd) of 9.5 as reported by Czyrny [14]), 71 persons per group are needed. Taking into account a 15% loss to follow-up, the required sample size is 82 persons per group (164 persons in total).

### Interventions/services

Supported Fast track multi-Trauma Rehabilitation Service

SFTRS involves the following:

a) The rehabilitation physician from the rehabilitation centre is routinely involved at a very early stage post-trauma. This allows an early start for multidisciplinary rehabilitation treatment;

b) Early transfer (within five days after having been added to the waiting list from the rehabilitation centre) to a centralised, specialised trauma rehabilitation unit equipped with facilities for early training programs;

c) Early individual rehabilitation goal setting;

d) Close co-operation and exchange of views and experiences between the trauma surgeon and the rehabilitation team by, for example, monthly clinical sessions and individual patient visits by the trauma surgeon in the first weeks after discharge;

e) Well-documented treatment protocols for multi-trauma patients for both the hospital and rehabilitation centre phases.

Three phases can be identified in the treatment of multi-trauma patients:

1 Early rehabilitation phase

2 Stage II rehabilitation phase

3 Discharge or post-discharge phase

#### - Phase 1: Early rehabilitation phase

In the early rehabilitation phase, the patient is not allowed to mobilise weight bearing. Consequently, the physiotherapist is concerned with maintaining joint mobility, muscle strength, sitting balance, condition and training transfers as well as treatments with non-weight-bearing conditions such as hydrotherapy and non-weight-bearing gait training. There are 10 sessions per week of 30 minutes each. In addition, fitness, gymnastics, table tennis, swimming, bowling, hand bike, wheelchair training, and archery are given. There are 2–3 sessions per week for each treatment modality of 60 minutes each. The occupational therapist advises on bed posture, mattress types, aids for independent daily self-care, wheelchair-dependency training and meaningful activities that can be performed while wheelchair-bound. In addition, the wheelchair accessibility and wheelchair friendliness of the patient's home are studied. If necessary, written and oral advice on temporary and long-term adaptations to the home is given and support is given and the patient is helped to apply for financial support so that the patient can return home as soon as possible. At first, this would be for a day or two at the weekend, supervised by an occupational therapist, but would later become permanent. With regard to work, the patient's job is analysed and the patient's workplace is visited. There are 4 sessions per week of 30 minutes each. The social worker and the psychologist will see every multi-trauma patient within the first week after admission. The social worker helps the patient to return home by dealing with the family and offering advice and support to the patient on financial matters, transport facilities. The social worker also contacts the employer and company doctor to look into the possibility of reintegrating the patient into their present job. The psychologist will examine the patient with regard to such conditions as mood disorders, posttraumatic stress syndrome (PTSS), acceptance problems and cognitive problems. The latter requires extensive neuropsychological testing. In addition, individual and group psychological counselling and specialised treatment for PTSS are given. If necessary, the rehabilitation specialist can refer the patient to a consultant in psychiatry, a consultant in neurology, a consultant in internal medicine, a consultant in rheumatology and/or a consultant in urology, who is affiliated to the rehabilitation centre.

#### - Phase 2: Stage II rehabilitation phase

In the Stage II rehabilitation phase, new treatment aims are added by the physiotherapist. These might include a gradual individual weight bearing scheme, co-ordination training and functional training. There are 7 therapy sessions per week of 30 minutes. In addition, fitness, gymnastics, table tennis, swimming, rowing, cycling and archery are given. This is offered in 2–4 sessions per week for each treatment modality of 60 minutes each.

The occupational therapist continues with the treatment goals as mentioned for phase I and trains the patient to perform household tasks, hobbies, etc in a home-like environment. There are three sessions per week of 30 minutes each. In addition, group therapies such as occupational therapy and recreational therapy are given 2–4 times per week each.

The social worker and the psychologist continue the work mentioned in phase I, depending on the individual needs of each patient.

#### - Phase 3: (Post) discharge phase

In the discharge phase, the patient is prepared for living at home and is referred to local physiotherapists, specialised sport clubs and mental health care professionals.

#### Conventional multi-trauma care service

CTCS is provided in several centres. Multi-trauma patients are admitted to hospital via the A&E department. After possible surgery, they are transferred to the IC-unit, followed by the hospital's nursing ward, where the patient may stay for several days or weeks. The trauma surgeon, as chief consultant, decides whether or not a rehabilitation physician will be consulted during hospitalisation. Next, ensuing treatment may take place in the hospital's outpatient clinic, in a rehabilitation centre, in a nursing home or with a local GP or physiotherapist. Van Vree and co-workers ([15]) reported that, typically, each of the CTCS "stages" might have its own more-or-less autonomous treatment perspective, depending on the professional's individual treatment views and experience.

The effectiveness of multi-trauma rehabilitation interventions and its constituting elements has been established in numerous studies. Recently, Holtslag [16], in his PhD research, investigated the long term outcome after major trauma. However, whereas most clinical studies compare single treatment outcome, no randomised clinical trials on treatment effects in multiple trauma patients have been found to date.

### Data collection

Baseline measurements will be administered as soon as possible post-injury (= T0). Further measurements will be taken at 3 months (= T1), 6 months (= T2), 9 months (= T3) and 12 months (= T4) post-trauma (see also flow chart, figure 1).

### Demographic and medical variables

Upon arrival at the A&E (= T0) the following variables will be recorded: age, gender, medical history, mediation usage, diagnosis, ISS score, date and time of trauma and Glasgow Coma Scale (GCS).

As soon as possible post-injury the following data are collected: trauma treatment, possible complications (description, number and extend) and time in hospital. The following additional data are recorded upon admission in the rehabilitation centre: individual rehabilitation treatment aims, pre-trauma psychosocial status and pre-trauma employment status.

### Outcome measures

Outcome measures are presented in table 2.

**Table 2:** Primary and secondary outcome measures

Primary outcome measures	Generic quality of life	SF-36
	Functional health status	FIM
Secondary outcome measures	Anxiety and depression	HADS
	Cognitive functioning	MMSE
	Extent to which individual ADL treatment goals are met	COPM

Note: Costs will be assessed using the PRODISQ, a cost questionnaire and hospital database data		

FIM: Functional Independence Measure; COPM: Canadian Occupational Performance Measure; HADS: Hospital Anxiety and Depression Scale; MMSE: Mini-Mental State Examination; PRODISQ: PROductivity and DISease Questionnaire

### Primary outcome measures

In the current study the primary outcome measures are FIM, measuring quality of life and SF-36, measuring functional health status. In several studies it was found that in multi-trauma patients quality of life and functional recovery do not solely depend on injury severity and complications [6-9], but also on psychological and social factors (e.g. [5,17-20]) as well as the patient's cognitive status [21]. The Functional Independence Measure (FIM) is widely used in assessing functional health status in different groups of patients. Baldry-Currens [22,23] recommended using the FIM in assessing trauma outcome, the FIM correlating high with measures of injury severity and demonstrating clinical and statistical significance. Similarly, Hetherington and co-workers [24,25] reported that in rehabilitation services, the FIM is a useful, practical and simple methodology, providing a measure for assessing the original disability, its progress and residual limitations. The National Trauma Data Bank collects data on trauma centre performance throughout the USA. As to functional outcome assessment in trauma patients FIM data are used [26].

At an international and interdisciplinary consensus conference in 1999 about the assessment and application of quality of life (QoL) measures after multiple trauma, experts clinicians and methodologists agreed on the SF-36 as generic tools for QoL assessment across all trauma patients [27]. In the proposed study both generic QoL and utilities will be derived using the SF-36. An overall utility score for population based QoL can be obtained, which facilitates comparisons with other interventions, i.e. the social tariff of the SF-36 [28-33].

### Secondary outcome measures

The Canadian Occupational Performance Measure (COPM) is an individualised client-oriented measure to assess the evolution of self-perception of skills in patients across time [34,35]. The COPM was, for example, used by Trombly and co-workers [36] to investigate the association between participation in goal-specific outpatient occupational therapy and improvement in self-identified goals in adults with acquired brain injury. In our study the COPM will be used to assess the extent to which individual treatment aims of the multi-trauma patient, set during rehabilitation, are met.

The Hospital Anxiety and Depression Scale (HADS) gives clinically meaningful results as a psychological screening tool, in clinical group comparisons and in correlational studies with several aspects of disease and quality of life. It is sensitive to changes both during the course of diseases and in response to psychotherapeutic and psychopharmacological intervention. Finally, HADS scores predict psychosocial and possibly also physical outcome [37]. The HADS has been used by Kempen and colleagues [38] to investigate the effect of depressive symptoms on the recovery of activities of daily living after fall-related injuries to the extremities in older persons. As stated before, anxiety and depression, among others, may influence therapy outcome in multi-trauma rehabilitation. Therefore, in the present study the HADS is used to assess this aspect. The Mini-Mental State Examination (MMSE) is a test that briefly surveys global mental status in a wide range of cognitive domains [39-41]. Jackson and co-workers [39] used the MMSE in trauma survivors without intracranial haemorrhage. Their findings corroborated earlier research stating that these patients display persistent cognitive impairment associated with functional defects, poor quality of life, and an inability to return to work [39]. In our study the MMSE will be used similarly, i.e. to assess global cognitive functioning of multi-trauma patients.

### Treatment credibility and expectancy

In studies comparing the effectiveness of different treatment regimes, differences in treatment credibility and expectancy may influence the outcome. In the proposed study the credibility/expectancy questionnaire (CEQ) [42] will be administered directly following the explanation of the study's rationale to patients, i.e. after informed consent has been obtained.

### Determination of costs

General considerations:

For the economic evaluation the main research question is:

From the viewpoint of the society is another organisation of professional care service for trauma patients (i.e. SFTRS) compared to CTCS preferable in terms of costs, effects and utilities?

Based on this main research question several sub-questions are relevant:

1) What are the costs of SFTRS compared to CTCS preferable in terms of costs, effects and utilities?

2) What are the extra effects (measured in quality of life, utilities, and saving by reducing inpatient hospital admissions of multi-trauma patient) of SFTRS compared to CTCS preferable in terms of costs, effects and utilities?

We hypothesise that SFTRS is associated with a reduction in health care and patient costs, and an improvement in quality of life, compared to CTCS. We expect SFTRS to be cost-effective from a societal perspective. Assessments of the quality of life and costs will take place at T1 through T4.

In the cost identification, the following costs are considered:

Health care costs: cost of the intervention program and other health care resources both by the patient and the caregiver.

• Patient and family costs: informal care, paid domestic help, transportation, over the counter medication, and other out-of-pocket expenses.

• Production losses: absenteeism, presenteeism (loss of productivity while at work), and compensation mechanisms for both the patient and the caregiver, if relevant.

Measurement of volumes:

• Hours spent on the intervention program will be recorded on a pre-structured form by the acting healthcare professionals.

• All other health care costs and patient & family costs will be recorded in a cost questionnaire.

• Production losses will be measured using the patient modules of the PRODISQ [43,44].

The PRODISQ will be used together with the costs questionnaire, every 3 months at baseline and T1 through T4.

For the valuation of health care costs and patient & family costs, an update of the Dutch manual for costing in economic evaluations [45] will be used. For care for which no costs-guidelines are available estimations of the costs will be made, based on the real costs and/or on population based estimates from literature. Valuation of production losses will be based on a modification of the friction cost method

Both generic Quality of life (QoL) and utilities are derived from the SF-36. An overall utility score for population based QoL can be obtained, which facilitates comparisons with other interventions, i.e. the social tariff of the SF-36 [28-33].

The primary outcome measure for the cost-effectiveness analysis will be FIM. The primary outcomes measure for the cost-utility measure will be utilities based on the SF-36 social tariff.

The time horizon is 12 months. Ratios will be determined, based on incremental costs and effects of SFTRS compared to CTCS. The cost-effectiveness ratio will be stated in terms of costs per improvement on the FIM. The cost-utility ratio will focus on the net cost per QALY gained. Bootstrap re-sampling techniques [46,47] are used to explore cost-effectiveness uncertainty.

Sensitivity analyses will be performed for the costs that turn out to have the largest impact on the differences in total costs between SFTRS and CTCS. In these analyses both the variance in volumes and prices will be considered. The range over which uncertain factors are thought to vary will be assessed by calculating a minimum and maximum (mean value of costs minus or plus the SD).

### Statistical analyses

In non-randomised comparative studies, variations in case mix between centres can influence the interpretation of outcome data [48]. Therefore, for each of the data sets collected at T1 through T4, differences in outcome variable between the 2 services will be tested using multiple MANCOVA's, entering various indicators of case mix as co-variates, i.e. age, gender, ISS, number of complications, pre-trauma psycho-social status.

When patients drop out of the study, the reason for their withdrawal will be recorded. Drop-outs may bias the treatment effect evaluations. Therefore, the following regime will be applied:

- Missing T4 measurement: 'last-observation-carried-forward' principle will be applied.

- Missing T3 or T2 measurement: linear interpolation of data using data from adjacent time points (e.g. T1 and T4) for imputation.

- Missing T0 or T1 measurement or more than 2 missing measurements: discarding of patient data and influx of additional patient in order to meet n = 82 per group.

## Discussion

The main objective of this study is to examine the effectiveness, the costs and the cost-effectiveness of an integrated Supported 'Fast Track' Rehabilitation Service for multi-trauma patients (SFTRS) involving dedicated early rehabilitation intervention programs.

As there are no publications found about contrasts in (cost-) effectiveness between different multi-trauma rehabilitation services it is important to investigate whether a new rehabilitation service is more effective than the conventional service.

During the conceptualisation of the study design several choices had to be made. The major ones will be discussed.

First, randomisation in this study is not possible because of practical and ethical reasons. The distance between the two centres is approximately 150 km. Since this study includes acute (complex) multi-trauma patients who need to be take care of as soon as possible it is not justified to transport patients for hours to accommodate to any randomisation procedure.

Second, in literature the 'severe multi-trauma' patients are frequently reported as having at least 2 or more injuries of which at least 1 is life threatening (ISS > = 16). However, during a pilot study it became apparent that part of the multi-trauma patients suffering from complex multiple injuries and who were admitted to the rehabilitation centre had an ISS < 16. Clinically speaking, it concerned patients suffering from one of the following injuries: a) complex multiple injuries on both lower extremities; b) a combination of one upper and one lower extremity injury, the latter of which could not be used in load-bearing; or c) complex pelvis/acetabulum fractures. Multi-trauma patients, who often suffer from skull and brain injuries, generally have an ISS > = 16. However, especially in complex pathology of the locomotor system, necessitating intensive rehabilitation training, the AIS system often scores less. Given the fact that these patients do participate in CTCS and SFTRS, indicating that they do belong to the target population in accordance with the other inclusion criteria, these patients have been added to the project population. This classification of multi-trauma patients is in accordance with the revised AIS coding ("AIS upgrading") which is currently being elaborated internationally.

In the present study the definition of multi-trauma reads: "Multi-trauma is defined as having at least 2 or more injuries of which at least 1 is life threatening, including a) trauma with ISS > = 16, b) complex multiple injuries on both lower extremities, c) a combination of one upper and one lower extremity injury, the latter of which can not be used in load-bearing, or d) complex pelvis/acetabulum fractures".

In conclusion, this paper describes the design of a prospective, multi-centre, non-randomised clinical trial that will investigate the (cost-) effectiveness of a new Supported Fast Track multi-Trauma Rehabilitation Service (SFTRS). The inclusion of the patients will start in October 2008 and will continue until May 2010. The results of this study will give evidence whether the new 'fast track' rehabilitation service for multi-trauma patients is more effective than the conventional care service, and thus should be introduced nation-wide.

## Competing interests

The authors declare that they have no competing interests.

## Authors' contributions

SK is the main researcher and has conceptualised this paper together with HS. HS has written the protocol for this study and is also the project leader of this study. BH, HS and PB originated the idea for the study. SE has co-written the study protocol regarding the cost aspects. HS and BH are the supervisors of SK. The promotor of SK is PB.
